# “*When Treatment Is More Challenging than the Disease*”: A Qualitative Study of MDR-TB Patient Retention

**DOI:** 10.1371/journal.pone.0150849

**Published:** 2016-03-09

**Authors:** Kalpita S. Shringarpure, Petros Isaakidis, Karuna D. Sagili, R. K. Baxi, Mrinalini Das, Amrita Daftary

**Affiliations:** 1 Department of Preventive Social Medicine, Medical College, Baroda, India; 2 Operational Research Unit, Médecins Sans Frontières, Mumbai, India; 3 The Union, South East Asia Office, New Delhi, India; 4 Operational Research Unit, Médecins Sans Frontières, Delhi, India; 5 McGill International TB Centre, McGill University, Montreal, Canada; 6 Centre for the AIDS Programme of Research in South Africa (CAPRISA), Nelson R. Mandela School of Medicine, University of Kwazulu Natal, Durban, South Africa; 7 Dalla Lana School of Public Health, University of Toronto, Toronto, Canada; Instituto de Diagnostico y Referencia Epidemiologicos, MEXICO

## Abstract

**Background:**

One-fifth of the patients on multidrug-resistant tuberculosis treatment at the Drug-Resistant-TB (DR-TB) Site in Gujarat are lost-to-follow-up(LFU).

**Objective:**

To understand patients’ and providers' perspectives on reasons for LFU and their suggestions to improve retention-in-care.

**Design:**

Qualitative study conducted between December 2013-March 2014, including in-depth interviews with LFU patients and DOT-providers, and a focus group discussion with DR-TB site supervisors. A thematic-network analysis approach was utilised.

**Results:**

Three sub-themes emerged: (i) Struggle with prolonged treatment; (ii) Strive against stigma and toward support; (iii) Divergent perceptions and practices. Daily injections, pill burden, DOT, migratory work, social problems, prior TB treatment, and adverse drugs effects were reported as major barriers to treatment adherence and retention-in-care by patients and providers. Some providers felt that despite their best efforts, LFU patients remain. Patient movements between private practitioners and traditional healers further influenced LFU.

**Conclusion:**

The study points to a need for repeated patient counselling and education, improved co-ordination between various tiers of providers engaged in DR-TB care, collaboration between the public, private and traditional practitioners, and promotion of social and economic support to help patients adhere to MDR-TB treatment and avoid LFU.

## Introduction

India ranks first among the 27 multidrug-resistant tuberculosis (MDR-TB) high burden countries worldwide, contributing to 21% of all estimated MDR-TB cases [1]. Monitoring MDR-TB outcomes, especially loss-to-follow-up (LFU), treatment failure and death,is critical for surveillance and the designof acomprehensive MDR-TB control program [2–4].

Studies have recorded high rates of LFU among MDR-TB patients, from 12 to 29% [3,5–9].LFU patients are more likely to die or develop more severe and resistant forms of TB [10].The reasons for LFU are varied and highly complex. Quantitative as well as qualitative research has shown that medical factors, such as adverse drug effects, addiction to illicit drugs; patient factors, such as gender, education status, income, and experiences with stigma; as well as health system factors, such patient-provider interactions, quality of care, and directly observed treatment (DOT), may impact adherence to MDR-TB treatment and trigger LFU [6,7,11].However, there is limited knowledge about how these issues may influence LFU and MDR-TB outcomes in the Indian context [12].

In the state of Gujarat, we found nearly 20% of MDR-TB patients are routinely LFU [5]. A recent analysis of retrospective data from 2010–2013 allowed our team to identify several negative associations between clinical and programmatic lacunae and patient LFU [5]. We believe there is a need to additionally examine social and behavioral factors associated with LFU, to develop more targeted strategies to support patients during treatment and reduce attrition from care [3,6,9,13].In this paper, we describe findings from a qualitative study aimed to characterize determinants of LFU among MDR-TB patients in Gujarat fromthe perspectives of patients and health care providers.

## Methods

### Ethical considerations

The study was approved by the Institutional Ethics Committee for Human Research (IECHR) at Baroda Medical College (Vadodara, India) and the Ethics Advisory Group of the International Union Against Tuberculosis and Lung Disease (Paris, France). Written informed consent was taken from each participant. Patient names were not collected in the study, and all participants were given the freedom to withdraw at any time during the interview. De-identified data was shared with the co-investigators for analysis. The study adhered to COREQ guidelines [14].

### Study Design

We used a qualitative study design to capture patient and provider experiences with MDR-TB treatment and follow up. Based on our preliminary fieldwork, we determined private in-depth interviews (IDIs) would be best suited to capture the personal experiences of patients and DOT providers, whereas a focus group discussion(FGD) would be more feasible for data collection from district drug-resistant TB (DR-TB) Supervisors.

### Study Setting/Area

The study was set at a DR-TB site in Baroda, Gujarat, which was established in February 2010 to initiate and monitor patients on MDR-TB treatment under the endorsement of the Indian Revised National TB Control Programme (RNTCP) [5].The site attends to patients from rural, urban, and tribal areas. Treatment is provided under a complete DOT (directly observed therapy) approach, including an intensive phase for the first 6 months, which may be extended to 9 months, followed by a continuation phase of 18 months. Patients are only admitted for the first 5–7 days at the point of MDR-TB treatment initiation.

### Study Population

LFU patients were defined as those patients whose treatment had been interrupted for two or more consecutive months, for any reason [2].Site records showed that since 2010, 153 patients had been LFU over a period of 4 years. A line-list of these LFU patients, including their socio-demographic characteristics, was obtained from the DR-TB Supervisors; patients who had moved residence or died post LFU were excluded. Of remaining LFU patients, approximately 25% (i.e., 36 patients) were selected purposively for IDIs to ensure adequate representation of age groups, sex, socio-economic status, district, and area of residence. Patients who were unwilling to participate, or not in a healthy state to be interviewed were excluded.

An FGD was organized among the DR-TB supervisors, who work for the RNTCP and assist the District TB Officer with MDR-TB logistics and in DOT supervision and monitoring of MDR-TB patients belonging to the primary health institution area. IDIs were organized with DOT providers. Supervisors and providers who were unwilling to participate or not available at the time of data collection were excluded.

### Data collection

Data was collected over a period of 4 months, from December 2013 to March 2014. A semi-structured interview questionnaire was used for patient IDIs and a semi-structured topic guide was used for provider IDIs and the FGD. All data was collected by a team of two members including the study PI (lead author), trained in qualitative interviewing, and a highly skilled note-taker, trained in pre-arranged shorthand, in the local vernacular (Gujarati), in a private place that was comfortable for participants. Interviews were not recorded in order to facilitate open and frank discussions. All patients, providers and FGD participants provided their written and informed consent to participate prior to data collection. Minors were accompanied by an adult during the interviews, who also consented to their participation. IDIs lasted 45–60 minutes while the FGD took about1.5 hours.

### Data Analysis

We utilized thematic network analysis as our framework for analysis, as described by Attride-Stirling [15].The interviewer (lead author) and note-taker transcribed, translated, and cross-checked all FGD and IDI data, as well as interviewer field notes that included non-verbal cues, immediately after data collection to ensure data credibility and enhance the reliability of our interpretations. Data was entered into WeftQDA0.9.4qualitative software.Two co-authors coded each interview independently using ad-hoc codes, which were repeatedly applied to the data set through continuous comparisons within and across the transcripts and field notes. This led to the development of more selective, or inductive, codes. Inductive codes were grounded in the study narratives [16,17]. Discrepancies in coding and re-coding were resolved by consensus, and led us toorganically identify several trends and patternsaround the central study theme of ‘Reasons for LFU’.

## Findings

### Participant characteristics

Of the 36 patients selected purposively for the IDIs, 23 were males. The age of the patients ranged from 13 years to 55 years. Among all patients, 14 were from rural areas, 10 from tribal areas and 8 from urban areas. Three patients had died when they were traced for the interview. One patient was unavailable to participate in the interview due to work. Therefore, IDIs were taken of 32 participants (23 males, 9 females), all of who consented to the study.

The FGD was attended by six DR-TB supervisors. IDIs were conducted with seven DOT providers, who included health care workers and practitioners.

**Fig 1:**
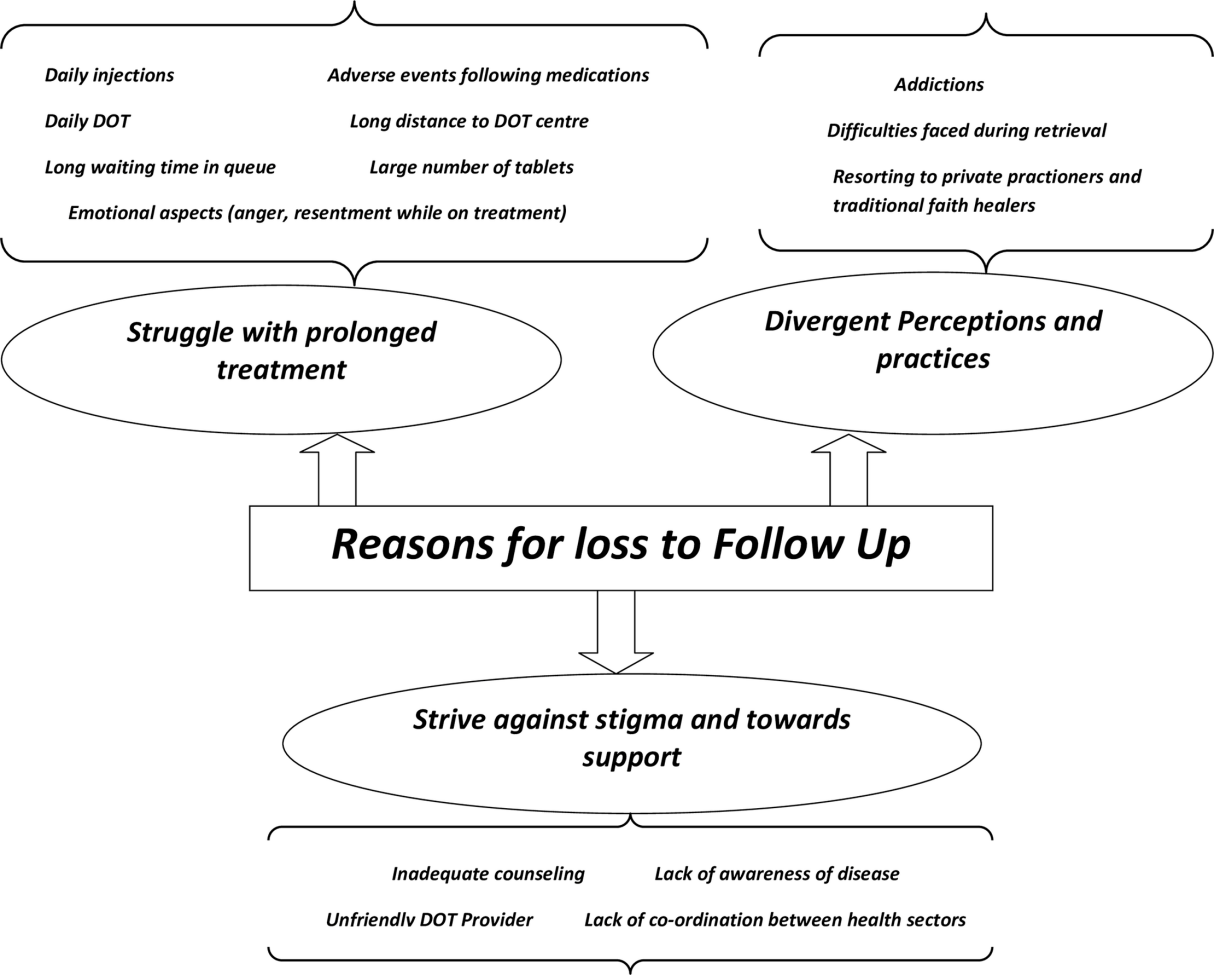
Thematic analysis for “Reasons for loss to follow up.

The overarching theme of our qualitative analysis, “Reasons for LFU”, was comprised of the following basic and organizing sub-themes: 1) Struggle with prolonged treatment; 2) Strive against stigma and toward support; and 3) Divergent perceptions and practices. Fig 1 illustrates our thematic analysis in a web-like, non-hierarchical manner.

### Struggle with prolonged treatment

Patient narratives pointed to a difficult journey with MDR-TB treatment. They reported daily injections, high pill burden, side effects, and the long duration of therapy to be major barriers to adherence. Here are the words of one patient who was frustrated that he was expected to keep adhering to such a long course of injectable treatment: *“I had taken it [i*.*e*., *medicines] properly for 7–8 months with injections but then stopped*. *How much should one take?”(P6*, *Male*, *33 years)*. Another patient had experienced severe discomfort during his treatment. He recalled discontinuing his medicines when he felt they made him lose interest in the things that he would have typically enjoyed: *“My whole body would ache*. *I used to get stools as hard as a camel’s heel [i*.*e*.,*constipated]*.*I could not eat*. *I did not find any food to be of good taste even though they [i*.*e*., *my relatives] prepared such good food for me*. *I left treatment*.*”(P1*, *Male*, *58 years)*

Providers also felt that the debilitating early adverse events following second-line TB agents, long treatment duration, and injectable drugs daunted patients, and inhibited adherence. But although most providers commiserated with their patients’ perspectives, they also firmly believed in the necessity of treatment.

*“The duration of treatment is of 2 years*. *And that too*, *with injections for the first 6–9 months*! *Even I cannot take treatment for so long*. *Patient gets admitted*, *starts treatment at the DR-TB Centre for a week*. *But after discharge*, *he gets to know that the duration of treatment is of two years*! *Even I cannot take*.*”(DOT Provider)*

*“Till the time adverse events and injection related issues do not decrease*, *retrieval [i*.*e*., *patient retention] will be difficult*.*”*(DOT Provider)

Long commutes to the DOT clinic and long waiting time in queue for receiving daily medicines were identified as key determinants of treatment interruption. Daily visits interfered with patients’ ability to sustain their routine job/livelihood. Adverse drug effects compelled many patients to take time off work, and suffer further losses in income. When perceived to be intolerable, adverse effects also led to treatment interruptions and discontinuation. Several patients migrated out to other areas for work, making it difficult for them to continue accessing treatment from the DR-TB site.

For example, one patient who had to endure a long daily for his treatment said, *“I took DOT for 2 months*, *stopped treatment due to long distance between provider and my house*.*”(P16*, *Male*, *27 years)*Another patient described how her medication side effects prevented her from attending to her household responsibilities: *“I felt sleepy the whole day*. *If I took [medicines] at lunch time*, *who would prepare food at home?”(P4*, *Female*, *52 years)*Similarly, another patient described the dilemma he faced trying to balance his medical needs with his financial obligations: *“I cannot afford to take treatment at daily cost of earning money*. *I have a family to feed*.*” (P18*, *Male 35 years)*This was echoed by the providers:*“Sometimes the patient have a moving job [i*.*e*., *migratory work]*. *He is the sole earning person of the family*.*”(DOT Provider)*

Providers also believed that patients’ prior history with (failed) TB treatment and/or non-adherence were possibly triggers for LFU during their present MDR-TB treatment. As one DOT provider stated, *“They are already fed up with other TB treatments taken before*.*”* Patients appeared to shift between feelings of rage and despair, as they dealt with their own frustration with prior episodes, prolonged treatment regimens and adverse drug effects, competing social and economic obligations, and persisting interference on the part of the health system from which they longed to escape. One patient was so disheartened by her long and painful treatment, no amount of counseling could convince her to continue taking her medications:*“I would throw away my medicines in anger*. *They smelt bad*.” She had suffered such a negative experience that even when prodded tore-start treatment for the sake of her young son, she said, *“Kids grow up*. *There will be someone [else] who will take care [of him]*.*” (P3*, *Female*, *35 years)* Faced with the multiple challenges of adverse drug effects, job loss, commuting difficulties, and household obligations, patients were prone to stop treatment the moment their symptoms began to resolve. As one patient stated, *“I took treatment for 3 months and felt better*, *so I left*.*” (P1*, *Male*, *58 years)*

Provider FGD and patient interviews revealed that retrieval action for LFU patients was generally very difficult:*“We try to bring back the patient back on treatment but it is difficult*. *One of the LFU patients told us*, *‘If you come to my house again*, *I will beat you*, *else I will commit suicide’*.*”(DOT Supervisor)*Although a few patients did re-initiated treatment subsequent to successful retrieval, many were again LFU due to migration, adverse drug effects, and social problems. For example, one patient who was keen to re-initiate treatment was unable to sustain his commitment because he could no longer rely on his relatives to set aside their time and responsibilities, and support him through the tumultuous regimen: *“Who will take care of me if I fall sick [i*.*e*., *from the medication side effects] again*? *Don't they [i*.*e*., *my family] have to earn a living?"(P29*, *M*, *23 years)*

### Strive against stigma and toward support

Stigma, lack of family support, the unfriendly attitude of DOT providers during treatment and retrieval action, and the lack of adequate counseling made it all the more difficult for patients to continue treatment, and in many cases led to LFU.

Although direct acts of discrimination were not reported, patients said they tried to keep their condition as secretive as possible. A diagnosis of MDR-TB could affect their social status within the community, particularly marriage prospects. This appeared to discourage adherence in some women, especially when they failed to receive the support of family members. As one adolescent female patient stated: *“My parents did not want me to take medicines as I am of marriageable age (I will not get a suitor)” (P14*, *Female*, *13 years)*.DOT providers too felt that along with adverse drug effects, lack of care and support from family was the main reason for LFU.*“There are two main reasons for LFU*. *Adverse events and lack of family support*.*” (DOT Provider)*

In some other patients, however, the fear of being labeled with TB appeared to promote adherence as they were keen to avoid a home visit by a DOT provider, one that could inadvertently expose them as a TB patient in their community. Home visits were routinely conducted as part of the retrieval action for non-adherent patients, but omitted among patients considered to be adherent. As one DOT provider stated: *“Those who do not want to be declared as having TB tell us not to come to their house*. *They are more regular in treatment as they fear that someone would come to their house if they did not take medicines*.*” (DOT Provider)*

Many patient participants reported having “unfriendly” or unhelpful DOT providers, which compounded their inclination to interrupt treatment. Some patients stopped treatment when they felt uninformed by their health providers about improvements in their health status and results from medical reports. *“The PHC people would collect sputum but not give me the report*. *So I left treatment*.*”(P18*, *Male*, *36 years)* Supervisors also admitted to the lack of consistent communication on the part of some DOT providers. This promoted LFU at crucial junctures when patients could have been retained in care with proper counseling and support. It was common that providers at more junior levels would refer patients to more senior providers. However, without proper follow through of such referrals, patients were likely to be LFU, as described in the narratives below:

*“We have to depend upon the “Health people”[i*.*e*., *counsellors*, *DOT providers] to do our work*. *We do not get much co-operation from them*.*Whenever the patients miss a dose the DOT Provider or PHC staff says that the DR-TB supervisor’s number is noted in [patient’s] file*. *Call them*. *The health staff do not take the responsibility of retrieving patients*.*” (DOT Supervisor)*

*“The Asha [i*.*e*.*community health workers] do not even realize that patient has missed her/his dose on a particular day*. *The only counselling which they receive is the initial health talk*.*” (DOT Supervisor)*

### Divergent perceptions and practices

The divergent circumstances surrounding a few patients led us to investigate several other factors affecting LFU, notably patient addictions, faith in traditional healers, engagement with private practitioners, and a patronizing attitude toward LFU patients.

Several patients reported suffering from addictions to tobacco or alcohol. These addictions appeared to interfere with their ability to adhere to prescribed MDR-TB therapy in addition to the challenges identified earlier:*“I am an alcoholic*. *I took both [i*.*e*., *treatment and alcohol]*. *Cough was relieved but I could not tolerate medicines [i*.*e*., *together with the alcohol]*, *so I stopped treatment*.*”(P21*, *Male*, *40 years)*Providers struggled with prioritizing adherence to MDR-TB treatment against the resolution of such co-morbidities, fearing patients would discontinue TB therapy if they were forced to give up their other ‘vices’: *“We cannot tell patients to leave addiction*. *If we tell them that*, *they will leave treatment*.*” (DOT Provider)*

Several patients also reported visiting traditional healers and ingesting alternative treatment during MDR-TB therapy, which in some cases led to their discontinuation from conventional treatment. *As one patient stated*: *“I don’t take treatment right now*. *After stopping treatment*, *we went to a religious place*. *There I got some Zaad-mool [i*.*e*., *tree roots] and took them for 15 days*. *[I]left the rest to God*. *Now my tests [sputum tests] are normal*.*”(P6*, *Male*, *33 years)* Patients’ faith in traditional healing practices and destiny were recognized as important deterrents to conventional care by both patient and provider participants.

Patients’ interactions with private providers also influenced their completion of MDR-TB treatment in the public health system, particularly when they were experiencing adverse effects which they were keen to be rid of. A few patients said they had stopped MDR-TB treatment on the order of their private doctors.

*“Private practitioner told me not to take the PHC [i*.*e*., *public health clinic] medicine as I was allergic to them*. *I was given a bed*, *saline and some injections at his clinic*. *I felt better in some time*.*” (P1*, *Male*, *58 years)*

*“I started private treatment and recovered within a month*. *Then I started taking these [i*.*e*., *MDR-TB medications]*. *After 3–4 months I could not eat*. *The family doctor [i*.*e*., *private doctor] said I had stomach swelling*. *He asked me to stop treatment*.*” (P4*, *Female*, *52 years)*

Provider participants shared that in some cases, LFU patients seen in the private sector were placed on regimens for drug-susceptible TB. With the reduced pill burden, patients gained a false assurance of being better: *“Patients taking MDR TB treatment from the government hospitals*, *go to private practitioners for treatment after LFU*. *The practitioners*, *most of the times give them Category I treatment [i*.*e*., *for drug-susceptible TB] and assure them that they will feel alright” (DOT Provider)*

A final subtle theme that emerged from study providers was a collective sentiment that despite their best efforts, patients failed to comply with providers’ advice. Providers strongly expressed that patients needed to listen to their health providers in order to get better.

*“Despite our best efforts [i*.*e*, *at retrieval]*, *some LFU remain so*.*”*

*“LFU (sighs in exasperation)*.*Those who will be will be*. *We do everything to help them*. *But some of them*, *they just do not listen*.*” (DOT Supervisor)*

*“These people [i*.*e*., *patients]*, *they do not listen*. *They bear the disease instead of bearing a few adverse events and the outcome will be ‘death’ most of the times*. *(DOT Provider)*

## Discussion

This study highlights the voices of patient and provider stakeholders attached to a major DR-TB centre in India, and offers a rich source of information about the ground reality for districts dealing with DR-TB in a high-burden, poorly explored region. While qualitative findings may not be easily generalizeable, this study is particularly useful given the paucity of contextualized data on MDR-TB in the Indian sub-context, and the global need to improve retention rates in MDR-TB care.

We have previously reported on clinical and programmatic factors driving LFU in this setting using quantitative methods [5].This qualitative study helped identify critical social drivers of LFU at the level of MDR-TB treatment (adverse drug effects, prolonged regimens, prior TB treatment), the patient (social support, financial stability, illness disclosure, fear of stigma, co-morbidities), and the health system (patient-provider communication and rapport, treatment counseling and literacy, and medical pluralism). Similar findings were identified in systematic reviews on adherence to MDR-TB treatment [6,7,11], in qualitative studies among MDR-TB/HIV co-infected patients in India [18,19], as well as in a mixed-methods study from Armenia [3].

Stigma is understood as contributing to LFU in MDR-TB [20–22].In our study too, stigma was seen to discourage treatment uptake and adherence. However, it encouraged some patients to complete treatment in order to avoid home visits by health personnel for retrieval action. While geographical location is not consistently found as a reason of LFU in the literature [23–27], we found distance from DOT sites could contribute to patient attrition in our setting. We also found that migratory work could promote LFU, as it escalated patients’ multiple challenges in relation to their mobile residence and socio-economic needs. Financial hardship has been identified as a major contributor to LFU in other studies [6,7,9–11,28], and migrant work has also been associated with poor adherence in TB [29].

While our study was restricted to the public health sector, patient narratives pointed to incongruence in prescribing practices between practitioners in the public and private sector. Some of our LFU patients, whose treatment was initiated at private clinics, were given drugs that they were already resistant to. The lack of knowledge or poor adherence to programmatic guidelines in TB care has been documented among private practitioners in the region [30]. However, patients’ willingness to adhere to the advice of their private doctors appeared to be an important driver of LFU, and warrants stronger collaborations between the public and private sector.

In our study, physical improvement after commencing treatment, or the lack of it, both appeared to result in some LFU. Patient education and counselling at the beginning of treatment as well as at periodic intervals is thus essential [28].This idea was also echoed in the FGD by the health providers. Given their high workload and that LFU patients do not immediately die, providers from the health sector did not realize the urgency of retrieval action at the critical juncture of symptom improvement, or the lack thereof. It is imperative that public health departments remain fully engaged in the routine monitoring and supervision of MDR-TB patients throughout their treatment course to devise the necessary retrieval action and support patients, given their difficult experiences with treatment [31].

Finally, our study found that MDR-TB patients may be subject to somewhat patronizing interactions within the health system.The idea that patients simply needed to “listen” to their providers was repeated in several interviews, suggesting a relatively paternalistic mindset to service provision (a phenomenon that is increasingly being recognized in the Indian health care system [32]), and the prioritization of rigid compliance over education and support. We believe that this important issue needs to be explored in subsequent studies.

Our study had some limitations. The sample was purposive and may not represent the full breadth of experiences among LFU patients and MDR-TB DOT Providers and Supervisors. While our decision to omit using audio-recorders allowed us to create an open environment and capture the most sensitive themes, we lost a degree of data richness. We countered this limitation by being fastidious with note taking during data collection, and document verbatim those narratives expected to be most relevant.

## Conclusion

We believe this study has important implications for service delivery in MDR-TB. First, accurate treatment education and counseling provided in a non-judgmental manner, that involves family members and garners their support from the outset, is likely to enhance patients’ commitment to treatment and reduce attrition. It is particularly imperative that we introduce these practices preemptively, before the onset of adverse drug effects, so patients and their family members are armed with correct knowledge before they become naturally inclined to discontinue treatment at the first sign of symptom improvement (or the lack thereof). It is also important that counseling be administered through the full course of treatment, as patients’ circumstances and experiences with medications are likely to change over the 1.5–2 years. Second, in relation to the above point, peer support groups may be effective ways to deliver emotional support and enhance treatment literacy, given that MDR-TB patients in India may lack access to emotional support and empathy within their own homes. The impact of social support and peer groups on LFU may be subsequently studied more objectively. Third, financial support by way of conditional grants or monetary incentives should be explored to encourage long-term retention in MDR-TB care, given patients’ dire socioeconomic circumstances and the public health imperative to reduce transmission of drug-resistant strains within the larger community. Nutrition and transport reimbursements have been associated with lower rates of non-adherence [27]. Fourth, there appears to be poor co-ordination and referral between primary health care level workers and Program staff, which may compound the difficulties patients already experience as they navigate the health system. This may be remediated through staff training, capacity building, and expanding communication channels between field DOT providers and district supervisors. Lastly, strengthening public-private partnerships, and communication with traditional healers, is a necessary component of TB service delivery in India given the high rate of medical pluralism.
